# Zuo Jin Wan Reverses the Resistance of Colorectal Cancer to Oxaliplatin by Regulating the MALAT1/miR-200s/JNK Signaling Pathway

**DOI:** 10.1155/2022/3032407

**Published:** 2022-10-07

**Authors:** Zhenzhen Wei, Jing Zhou, Hao Yu, Yunzhou Pu, Yuelei Cheng, Yi Zhang, Qing Ji, Huirong Zhu

**Affiliations:** ^1^Department of Medical Oncology & Cancer Institute of Integrative Medicine, Shuguang Hospital, Shanghai University of Traditional Chinese Medicine, Shanghai 201203, China; ^2^Medical Experiment Center, Jiading Branch of Shanghai General Hospital, Shanghai Jiao Tong University School of Medicine, Shanghai 201803, China

## Abstract

**Background:**

Oxaliplatin (L-OHP) is a common chemotherapy drug used in the treatment of colorectal cancer (CRC). Our previous work showed that Zuo Jin Wan (ZJW), a traditional Chinese medicine prescription, could improve sensitivity to L-OHP in the treatment of CRC, but the detailed mechanism is not clear. In previous mechanistic studies, we found that the miR-200s expression in CRC is associated with L-OHP sensitivity through regulation of MDR1/p-gp and the downstream c-JunN-terminal kinase (JNK) signaling pathway. Moreover, lncRNA-MALAT1 offers great potential in the regulation of drug resistance by interacting with miR-200s. Therefore, in this work, we explored whether ZJW could reverse L-OHP resistance in CRC by regulating MALAT1, miR-200s, and the downstream signaling pathway.

**Methods:**

Cell Counting Kit-8 and flow cytometry were used to detect the effects of ZJW combined with L-OHP on chemotherapy tolerance and cell apoptosis of HCT116/L-OHP cells. Western blotting and quantitative real-time PCR (qRT-PCR) were used to detect the activation of the JNK signaling pathway and the protein and mRNA expression levels of the drug resistance-related MDR1/ABCB1 gene in HCT116/L-OHP cells treated with ZJW. The binding sites of MALAT1 and miR-200s were predicted by bioinformatics tools and confirmed by qRT-PCR. qRT-PCR was used to detect the expression of miR-200s and MALAT1 in HCT116/L-OHP cells treated with ZJW. A xenograft model of CRC in nude mice was established to observe the effect of ZJW combined with L-OHP on the growth of subcutaneously transplanted tumors. Apoptosis in tumor cells was detected by TUNEL staining. The activation of the JNK signaling pathway and the expression of drug resistance-related proteins were detected by immunohistochemistry and immunofluorescence. qRT-PCR was used to detect the expression of miR-200s and the MALAT1 gene in the tumors.

**Results:**

Our study showed that ZJW could significantly decrease the proliferation and promote apoptosis of HCT116/L-OHP cells treated with L-OHP. We further proved that ZJW could reverse the drug resistance of HCT116/L-OHP cells by reducing MALAT1, indirectly upregulating miR-200s, alleviating the activation of the JNK signaling axis, and downregulating the expression of resistance proteins such as MDR1/ABCB1 and ABCG2. ZJW combined with L-OHP inhibited the growth of subcutaneously transplanted tumors and induced apoptosis in nude mice. ZJW reduced the expression of MALAT1 and upregulated the expression of miR-200s in transplanted tumors. In addition, ZJW also alleviated the activation of the JNK signaling pathway while reducing the expression of MDR1/ABCB1 and ABCG2.

**Conclusions:**

Our study identified that MALAT1 promotes colorectal cancer resistance to oxaliplatin by reducing the miR-200s expression. ZJW may reverse chemoresistance by inhibiting the expression of MALAT1 and regulating the miR-200s/JNK pathway, providing an experimental basis for the clinical application of ZJW in relieving chemotherapy resistance.

## 1. Introduction

Colorectal cancer (CRC) is one of the most common malignant tumors worldwide [1], with increasing incidence each year [2]. Oxaliplatin (L-OHP)-based chemotherapy improves the survival of CRC patients [3, 4]. However, the occurrence of drug resistance seriously damages the efficiency of chemotherapy [5, 6]. It has been reported that the response rate for L-OHP is 6%–31% and the median progression-free survival is 3.1–7 months [7]. Chemoresistance contributes greatly to treatment failure [8]. Therefore, it is urgent to develop effective strategies for enhancing the sensitivity of L-OHP to improve the survival and prognosis of patients with CRC. Traditional Chinese medicine (TCM) and its bioactive substances have been confirmed to improve treatment efficiency and prolong the survival of patients with tumors [9]. Zuo Jin Wan (ZJW) is a TCM prescription that has been widely used in gastrointestinal diseases. Our previous study demonstrated that ZJW could enhance the sensitivity to cisplatin and L-OHP in HCT116/L-OHP cells [10]. However, the mechanism by which ZJW reverses chemoresistance remains unknown.

Considerable efforts have been made to elucidate the mechanism of platinum resistance through biochemical characterization and molecular aspects, including reduced platinum accumulation, enhanced DNA repair, decreased apoptosis, and inactivation by thiol-containing biomolecules, including glutathione [11]. P-gp, ATP-binding cassette (ABC) drug transporter noncoding RNA, and the microbiome were confirmed to regulate the molecular and biochemical processes of chemoresistance [12, 13]. P-gp acts as an ATP-dependent outflow pump which can pump a variety of drugs out of cells to prevent intracellular accumulation [14]. miRNAs regulate the expression of mRNA by inhibiting the translation or stability of mRNA [15]. Long noncoding RNAs (lncRNAs) are capable of interacting with miRNAs by acting as competing endogenous RNAs. The dysregulation of lncRNAs/miRNAs is associated with the development of cancer [16]. MALAT1 is a lncRNA distributed in the nucleus that is highly expressed in CRC, liver cancer, breast cancer, and other cancer tissues [17]. The c-JunN-terminal kinase (JNK) signaling pathway contributes to drug resistance by regulating transporters and blocking the cell cycle and cell apoptosis [18]. Our previous studies have found that the overexpression of miR-200c can inhibit the expression of MDR1/P-gp and deactivate the JNK pathway to increase the sensitivity of drug-resistant human CRC cells to L-OHP.

Combined with previous research results, we speculate that ZJW reverses the drug resistance of CRC by regulating the MALAT1/miR-200s/JNK signaling axis. Thus, in this work, we investigated and confirmed the function of MALAT1 with respect to miR-200c through bioinformatics analysis and in vitro experiments. Using L-OHP-resistant cell lines and a xenograft model of CRC, we demonstrated the effect and the molecular mechanisms of ZJW in reversing chemoresistance in CRC. Overall, our study revealed the use of ZJW as a potential strategy for enhancing the antitumor efficacy of L-OHP in CRC treatment.

## 2. Methods

### 2.1. Preparation of the ZJW Extracts

ZJW is composed of *Rhizoma coptidis* and Evodia in the proportion of 6 : 1. All Chinese herbal medicines were purchased from the traditional Chinese medicine store (Shang, China) of Shuguang Hospital affiliated with Shanghai University of Traditional Chinese medicine. The mixture (70 g) was refluxed and extracted twice with ethanol (1 : 8, v/v) for 1 hour each time. The filtrate was concentrated and dried under vacuum at −80°C, and the dry powder yield was 21.2%. The powder was stored at 4°C. The preparation was standardized and quality-controlled according to the standards set by the State Food and Drug Administration (SFDA). One gram of the ZJW powder was accurately weighed by an electronic balance and dissolved in 1 mL DMSO. The samples were vortexed and sonicated overnight and sterilized by ultraviolet irradiation to prepare 1 g/mL ZJW. In the in vitro experiment, ZJW was diluted with medium to three concentrations: low (5 *μ*g/mL), medium (10 *μ*g/mL), and high (20 *μ*g/mL).

### 2.2. Cell Culture

Human colorectal cancer HCT116 parental cells were purchased from Shanghai Fine Cell Bank (Shanghai, China). The HCT116/L-OHP multidrug-resistant cell line was established and maintained in our laboratory. The HCT116 cell line was cultured in a RPMI 1640 conditioned medium (HyClone, China) containing 10% (v/v) heat-inactivated fetal bovine serum, 100 units/mL penicillin, and 100 *μ*g/mL streptomycin. HCT116/L-OHP cells were routinely cultured in a conditioned medium containing 5 *μ*g/mL L-OHP (Sanofi, France). The culture conditions were 37°C and 5% CO_2_ saturation humidity.

### 2.3. Animals and Xenograft Model

A total of 36 male nude mice of SPF cleanliness class, aged 8 to 12 weeks, were purchased from Shanghai SLAC Laboratory Animal Co., Ltd. (Shanghai, China, license no. SCXK2017-0005) and kept under specific pathogen-free conditions. The room temperature was 20°C, and the relative humidity was 60%. The 12 h cycle of light and shade was maintained, with freely available drinking water, and fasting was ensured for 12 hours before the experiment. The animal experiment was performed and approved by the Animal Ethics Committee of Shanghai University of Traditional Chinese Medicine. All experimental mice were tested in accordance with animal ethics standards and in line with the provisions and general recommendations of the regulations of the Administration of Experimental Animals of China. HCT116/L-OHP cells were grown in a medium, digested, separated by trypsin, washed, and resuspended in Hanks' solution (HBSS). There were 1 × 10^6^ HCT116/L-OHP cells in the logarithmic phase per 0.2 mL of HBSS. When the average size of the tumor reached 100 mm^3^, the mice were randomly divided into 6 groups (*n* = 5 in each group). The mice in the first group were treated with distilled water every day as the control group. The mice in groups 2-5 were intraperitoneally injected with L-OHP once every two days, and the injection dose (5 mg/kg) was half of the MTD of L-OHP, as mentioned earlier. The mice in group 3, group 4, and group 5 were given ZJW by intragastric administration at doses of 1027.5 mg/kg, 2055 mg/kg, and 4110 mg/kg, respectively. The mice in the sixth group were only given intragastric administration of ZJW at a dose of 1027.5 mg/kg to eliminate the toxicity of evodiamine. The length (*A*) and width (*B*) of tumors were recorded every 2 days, and the tumor volume was estimated according to the following formula: V = *π*/6 × *A* *∗* *B* *∗* *B*. The tumor growth map was generated according to the tumor volume and time. On the 28th day after treatment, the mice were killed and the tumor tissue was removed and weighed.

### 2.4. Cell Viability Assays

The oxaliplatin-resistant human colorectal cancer cell line HCT116/L-OHP in the logarithmic growth phase was selected, and the cell concentration was adjusted to 1 × 10^4^ mL. A total of 100 *μ*L per well was inoculated in a 96-well cell culture plate at 37°C in a 5% CO_2_ saturated humidity incubator. After 3–5 hours, when the cells adhered to the wall, the culture medium was discarded. Compared with the blank group, L-OHP and L-OHP combined with different concentrations of ZJW were added. After 48 hours of culture, 100 *μ*L of CCK-8 solution (Dojindo, Japan) was added to each well and then incubated in the incubator for 4 hours. The OD value (450 nm) was measured by an enzyme labeling instrument and the growth inhibition rate of the cells was calculated.

### 2.5. Analysis of Apoptosis by Flow Cytometry

The cells were inoculated in 6-well plates (4 × 10^5^/well). Twelve hours later, compared with the blank group, L-OHP and L-OHP combined with different concentrations of ZJW were added (the IC10 of ZJW in the CCK-8 assay was obtained). After 48 hours of incubation, the cells were digested by trypsin, washed twice with PBS, and counted. According to the instructions, 1× binding Buffer in the apoptosis kit (BD Pharmingen, USA) was used to produce a 1 × 10^6^ mL cell suspension. A total of 300 *μ*L of cell suspension was added to a new flow tube, and 5 *μ*L Annexin V-FITC and 5 *μ*L propidium iodide (PI) were added. After gently mixing, the reaction proceeded for 15 min at room temperature with light avoidance, 200 *μ*L 1× binding buffer was added to each tube, and the results were detected on the computer within 1 hour. Flow cytometry was used to detect apoptosis by determining the relative number of Annexin V-FITC-positive and PI-negative cells. Nonstained cells, cells stained with Annexin V-FITC alone, and cells stained with PI alone were used as controls. Monochromatic cells were used to regulate electronic compensation in the FL1 and FL2 channels.

### 2.6. Analysis of Apoptosis by TUNEL Assay In Vivo

The excised tumor was washed with xylene twice, each time for 5 min. The cells were rinsed once with gradient ethanol (100%, 95%, 90%, 80%, 70%) and twice with PBS. Using Proteinase K working liquid, the tissue was treated for 15–30 min and the TUNEL reaction mixture was prepared according to the instructions. The specimens were then covered with glass slides or sealing film in a dark wet box at 37°C for 60 minutes, then rinsed 3 times with PBS. Then, 50 *μ*L of converter-POD was added to the specimen after the slide was dried, and the slide or seal film was covered and reacted at 37°C in a dark wet box and rinsed 3 times within 30 minutes. Next, 50 *μ*L DAB substrate was added to the tissue, which was reacted at 25°C and then rinsed with PBS for 10 minutes. After image acquisition, the tissue was stained with hematoxylin or methyl green and immediately rinsed with water, then subjected to gradient alcohol dehydration, xylene for transparency, and neutral gum sealing. A drop of PBS or glycerin was added to the field of vision, and cell counts and images were acquired with an optical microscope.

### 2.7. Western Blot Analysis

HCT116/L-OHP cells in the logarithmic growth period were used to establish the control group and groups of different doses of ZJW. L-OHP was combined with different concentrations of ZJW (0, 10, 15, and 20 *μ*g/mL) for 48 h. The protein was extracted and quantified by a BCA protein assay kit (Beyotime, China), and the protein samples were treated. A 10% SDS-PAGE gel, sample protein, and marker (Thermo, China) were prepared. The proteins were then transferred to PVDF membranes after electrophoresis and sealed at room temperature for 2 hours. The closed membrane was immersed in the primary antibody and incubated overnight at 4°C. The next day, TBST was used to wash the film three times, each time for 10 min, and the membrane was soaked in the secondary antibody and incubated at room temperature for 2 hours. After washing the film, it was colored with ECL chemiluminescence solution (Cytiva, China), and the gray value was analyzed by ImageJ software. The primary antibodies used were MDR1/ABCB1 (CST, USA), ABCG2 (CST, USA), MRP4/ABCC4 (CST, USA), JNK2 (CST, USA), phospho-SAPK/JNK (CST, USA), and GAPDH (CST, USA). The secondary antibody used was HRP-labeled goat anti-rabbit/mouse IgG (*H* + *L*) (Beyotime, China). Each band was quantitatively analyzed using ImageJ software and normalized to the expression of GAPDH in the same lane.

### 2.8. Quantitative Real-Time PCR

RNA was extracted from cultured cells with TRIzol reagent (Takara, China). Total RNA was reverse-transcribed into cDNA according to the instructions of the reverse transcription kit (TIANGEN, China). An appropriate amount of cDNA was used as the template for PCR and amplified according to the qRT-PCR specifications (TIANGEN, China). Finally, the gene expression was analyzed with GAPDH and U6 as internal references, and relative expression levels were calculated by using the 2^−△△Ct^ method. PCR was performed using an ABI 7500 instrument (Applied Biosystems, USA). The primers used for real-time PCR analysis are listed in Table 1.

### 2.9. Immunohistological Analysis

The paraffin sections were incubated in the sealing solution (10% donkey serum + 5% skim milk + 4% BSA + 0.1% Triton X-100) for 10 minutes; then, hydrated sections were incubated with antibodies at 4°C overnight. After rinsing with phosphate-buffered saline (PBS), the slices were incubated with a diluted biotinylated secondary antibody for 30 minutes. Subsequently, the slides were washed with PBS again and incubated with the prefabricated avidin peroxidase macromolecular complex for 30 minutes. The peroxidase reaction was completed by incubation in PBS containing 0.01% hydrogen peroxide at room temperature for approximately 5 minutes. The slices were thoroughly washed in tap water, anti-stained in hematoxylin, dehydrated in anhydrous ethanol, deparaffinized in xylene, and examined under a microscope in synthetic resin.

### 2.10. Immunofluorescence Analysis

Frozen tissue sections were fixed in 4% paraformaldehyde for 10 min at room temperature and then washed twice with PBS. Blocking buffer (DakoCytomation, Glostrup, Denmark) was added for 30 min, and samples were then stained with primary antibodies and FITC-conjugated goat anti-rabbit IgG (Millipore). Sections were imaged using a TCS SP2 spectral confocal system (Leica, Germany). All experiments were conducted according to instructions from the antibody manufacturer.

### 2.11. Database Prediction

The starBase database is a widely used open-source platform for studying lncRNA interactions from CLIP-seq, degradome-seq, and RNA-RNA interactome data. Herein, the starBase database was introduced to analyze the expression correlation between miRNAs and genes or pseudogenes. Taking miR-200s as the target, the biological information in the lncRNA online database (https://starbase.sysu.edu.cn/browseNcRNA.php) should be used to predict the lncRNA molecules that may regulate miR-200s and select them according to the scoring results and related literature.

### 2.12. Statistical Analysis

SPSS 26.0 statistical software was used for analysis. The measurement data are represented by $\bar{x}$ ± *s*, and the *t* test was used for comparisons between the two groups. *P* < 0.05 indicates that the difference is statistically significant.

## 3. Results

### 3.1. Effect of ZJW on the Proliferation and Apoptosis of HCT116/L-OHP Cells

To avoid the effect of the cytotoxicity of ZJW on the inhibition of CRC cell proliferation, we detected the cytotoxic effect of ZJW on HCT116/L-OHP cells. The results showed that the IC_10_ dosage in HCT116/L-OHP cells was 20 *μ*g/mL (Figure (a)1). Below this dose, there was no significant difference in cell survival between treated cells and untreated cells. Therefore, in all cell proliferation experiments, ZJW was used to treat cells in the concentration range of 20 *μ*g/mL.

The CCK-8 results showed that HCT116/L-OHP cells were more resistant to oxaliplatin than sensitive HCT116 cells (Figure (b)1). When HCT116/L-OHP cells were treated with different concentrations of oxaliplatin for 48 hours, the inhibitory effect of oxaliplatin on HCT116/L-OHP cells was not obvious, but the inhibitory effect of ZJW combined with oxaliplatin on HCT116/L-OHP cells was significantly stronger than that of oxaliplatin alone (Figure (c)1). These results suggest that ZJW can enhance the sensitivity of HCT116/L-OHP cells to oxaliplatin in vitro.

To further explore the mechanism by which ZJW increases cell apoptosis, Annexin V and PI double staining was used to observe the changes in apoptosis induced by ZJW combined with oxaliplatin. The results showed that compared with the control group, the apoptosis rate of HCT116/L-OHP cells treated with oxaliplatin was 26% (Figures (d)1 and 1(e)). After intervention with different concentrations of ZJW combined with oxaliplatin, the apoptosis rate of HCT116/L-OHP cells increased to 36.2%, 52.8%, and 71.4%, respectively (Figures (d)1 and 1(e)), suggesting that ZJW can enhance the apoptosis of oxaliplatin-treated HCT116/L-OHP cells in a concentration-dependent manner.

### 3.2. ZJW Mediates the miR-200s/JNK Signaling Pathway to Regulate Drug Resistance in CRC

Our previous studies showed that the overexpression of miR-200c inhibits the activation of the JNK signaling pathway and reverses tumor drug resistance. Therefore, we detected whether ZJW affects the expression level of the JNK signaling pathway indirectly by affecting the expression of miR-200s. The qRT-PCR results showed that the mRNA expression of miR-200s in HCT116/L-OHP cells was significantly lower than that in sensitive cells. The mRNA expression levels of miR-200s in HCT116/L-OHP cells treated with L-OHP and different concentrations of ZJW were significantly higher than that in the control group (Figure (a)2). At the same time, qRT-PCR and Western blot showed that compared with the control group, the relative protein expression of JNK in HCT116/L-OHP cells did not change, while the relative protein expression of p-JNK decreased after ZJW intervention, suggesting that the JNK signaling pathway was inactivated (Figures (b)2 and 2(c)). These results suggest that ZJW may regulate the drug resistance of CRC by increasing the miR-200s expression and inhibiting the JNK signaling pathway in vitro.

MDR1/ABCB1, MRP4/ABCC4, and ABCG2 are all ABC transporter proteins associated with drug transport. We detected the expression levels of these proteins. The results showed that compared with sensitive cells, the relative expression of MRP4/ABCC4 protein in HCT116/L-OHP cells exhibited no significant change. The relative expression of MDR1/ABCB1 and ABCG2 proteins was increased, suggesting that the mechanism of drug resistance may be related to drug efflux on the cell membrane (Figure (a)3). After treatment with different concentrations of ZJW, the expression levels of MDR1/ABCB1 and ABCG2 resistance proteins in the low-, middle-, and high-dose ZJW intervention groups were significantly decreased compared with those in the control group (Figures (a)3 and 3(b)). In addition, compared with sensitive cells, the relative mRNA expression of MDR1/ABCB1 and ABCG2 in HCT116/L-OHP cells increased, while the relative mRNA expression of MDR1/ABCB1 and ABCG2 decreased in a dose-dependent manner after intervention with different concentrations of ZJW and the difference was statistically significant (Figure (c)3). These results suggest that the reversal effect of ZJW on drug resistance may be related to inhibiting the expression of MDR1/ABCB1 and ABCG2.

### 3.3. ZJW Regulates the MALAT1/miR-200s/JNK Signaling Pathway to Reverse Drug Resistance In Vitro

To study the existence of upstream regulatory genes in miR-200s, we used bioinformatics analyses to predict the existence of multiple complementary binding sites between MALAT1 and miR-200s (Figures (a)4 and 4(b)). Then, we verified the expression of MALAT1 and miR-200s in cells. We found that compared with sensitive cells, the relative mRNA expression of MALAT1 in HCT116/L-OHP cells was increased, while the relative mRNA expression of miR-200s was decreased, and the difference was statistically significant (Figures (a)2 and 4(c)).

To further study the internal relationship between miR-200s and MALAT1, we constructed a siRNA against the MALAT1 gene. The application of siRNA-MALAT1 resulted in a significant decrease in MALAT1 mRNA expression in HCT116/L-OHP cells (Figure (e)4). Verified by qRT-PCR, the relative mRNA expression of miR-200s was increased in HCT116/L-OHP cells transfected with siRNA-MALAT1 (Figure (f)4). In addition, the use of ZJW reduced the MALAT1 expression in HCT116/L-OHP cells (Figure (d)4). Therefore, we proved the function of MALAT1 in indirectly regulating the miR-200s expression. Combined with the abovementioned results, we suggest that ZJW may reverse chemoresistance in CRC by regulating the MALAT1/miR-200s/JNK signaling pathway.

### 3.4. ZJW Reverses Chemotherapy Resistance in CRC in a Nude Mouse Xenograft Model

We used HCT116/L-OHP cells to inoculate nude mice to build a xenograft model. After successful modeling, the nude mice were divided into 6 groups in order to observe the tumor volume and body weight. With the passage of time, the tumor volumes of nude mice in the model group and experimental group gradually increased. After 28 days of the administration, there was no significant difference in tumor size between the L-OHP group and the model group, but the combined administration of L-OHP and ZJW could significantly inhibit tumor growth, and the shrinkage of tumor volume in mice was concentration-dependent (Figures (a)5–5(d)). These experiments provide evidence that ZJW enhances drug sensitivity during chemotherapy in vivo.

To explore whether ZJW can also induce apoptosis in vivo, a TUNEL assay was used to observe the changes in subcutaneously transplanted tumors in nude mice. The results showed that compared with the normal saline control group, there was no significant change in apoptosis in subcutaneous tumor tissues of nude mice treated with L-OHP or ZJW alone. After intervention with ZJW combined with L-OHP, the expression of TUNEL in subcutaneous tumor tissue of nude mice increased significantly, and the difference was statistically significant (Figures (e)5 and 5(f)). These results suggest that ZJW can enhance the effect of L-OHP on the apoptosis of HCT-116/L-OHP cells in vivo, which is consistent with the in vitro experimental results.

### 3.5. Effects of ZJW on the MALAT1/miR-200s/JNK Signaling Pathway In Vivo

The immunohistochemical results showed that the relative protein expression levels of p-JNK in the treatment groups with L-OHP combined with different concentrations of ZJW were lower than that in the control group (Figures (a)6 and 6(d)). The Western blot results were consistent with the immunohistochemistry results (Figures (b)6 and 6(c)). These results showed that ZJW could regulate the drug resistance of CRC by inhibiting the JNK signaling pathway in vivo. qRT-PCR detection showed that, in accordance with the in vitro results, the relative mRNA expression of miR-200s was significantly upregulated (Figure (e)6). The relative mRNA expression of MALAT1 was significantly decreased in the treatment groups with L-OHP combined with different concentrations of ZJW compared with the control group in vivo (Figure (f)6).

The expression of ABCG2 was detected by immunohistochemistry, while the expression of MDR1/ABCB1 and MRP4/ABCC4 was detected by immunofluorescence to evaluate the effect of ZJW in vivo. In accordance with the results of the in vitro experiment, the expression levels of MDR1/ABCB1 and ABCG2 in the treatment groups with L-OHP combined with different concentrations of ZJW were lower than that in the normal saline control group (Figures (a)7 and 7(b)), but there was no significant change in the MRP4/ABCC4 expression (Figures (c)7 and 7(d)). Furthermore, Western blotting was used to verify the results. The relative protein expression of MDR1/ABCB1 and ABCG2 in the treatment groups with L-OHP combined with different concentrations of ZJW was significantly lower than that in the control group (Figures (e)7 and 7(f)). The qRT-PCR results coincided with the WB results (Figure (g)7). There was no significant change in the protein or mRNA expression of MRP4/ABCC4. These results suggest that ZJW may increase the sensitivity of CRC to L-OHP by decreasing the MDR1/ABCB1 and ABCG2 expressions. In summary, we proved that ZJW could increase the sensitivity to L-OHP, and the mechanism may be related to the downregulation of MALAT1/miR-200s/JNK signaling.

## 4. Discussion

Chemotherapy is currently still one of the main nonsurgical approaches to impede tumor progression in CRC patients with advanced stages. The occurrence of drug resistance and side effects limits the response rate of CRC patients to chemotherapy. The mechanisms of chemoresistance are complex. Plant-based phytochemicals are large parts of TCM, which are now used to treat various physiological diseases. Recently, herbal medicine has gained preference owing to its purity, strength, and cost-effectiveness [19]. Natural TCM compounds slow the spread of cancer by promoting apoptosis and inhibiting metastasis [20]. ZJW is a common prescription for gastrointestinal diseases such as gastritis, ulcerative colitis, and colorectal cancer. Our study demonstrated that ZJW could reverse L-OHP resistance in CRC in vitro and in vivo. Meanwhile, bioactive components such as berberine and evodiamine have been demonstrated to inhibit the growth of tumors and reverse chemotherapy resistance in multiple tumors [21, 22]. The combination of berberine and evodiamine displays higher anticancer activity while reducing side effects in CRC cell lines [23].

In recent years, it has been found that miRNAs participate in chemoresistance by affecting drug efflux, cell apoptosis, and cell cycle arrest [24]. Liu found that exosomes derived from cisplatin-resistant oral squamous cell carcinoma cells can transfer miR-21 to sensitive cells and induce cisplatin resistance [25]. It is widely reported that miR-200c is involved in colon cancer progression. It has been reported that the overexpression of miR-200c inhibits the proliferation of colon cancer by targeting the FUT4/Wnt/*β*-catenin pathway [26]. Our study showed that miR-200c overexpression in CRC could inhibit the activation of the downstream JNK signaling pathway and reverse drug resistance. The addition of ZJW increased miR-200s and inhibited the activation of the JNK pathway.

MALAT1 was one of the earliest discovered lncRNAs related to human diseases, and the high expression of MALAT1 predicts poor outcomes for CRC patients [27]. The expression level of MALAT1 is significantly increased in CRC patients and is related to advanced TNM stage, lymph node metastasis, and short survival [28]. MALAT1 has been proven to promote CRC cell proliferation and invasion by sponging miR-508-5p and enhancing RAB14 expression [29]. In addition, the expression of MALAT1 is prognostic for CRC patients treated with L-OHP. A high MALAT1 expression is associated with poor response to oxaliplatin-based chemotherapy and reduced survival in advanced CRC patients [30]. We found that MALAT1 was of great importance in the expression of miR-200s, and ZJW could regulate the expression of MALAT1 in drug-resistant CRC.

## 5. Conclusion

Our study identified that MALAT1 promotes colorectal cancer oxaliplatin resistance by reducing the miR-200s expression, and ZJW may reverse chemoresistance by inhibiting the expression of MALAT1 and regulating the miR-200s/JNK pathway. Thus, this study provides an experimental basis for the clinical application of ZJW to alleviate chemoresistance and improve the prognosis of CRC patients.

## 6. Disclosure

Zhenzhen Wei and Jing Zhou are co-first authors.

## Figures and Tables

**Figure 1:**
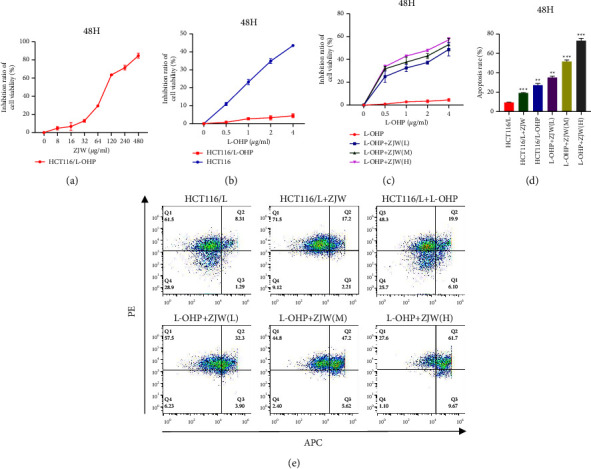
Effect of ZJW on the proliferation and apoptosis of HCT116/L-OHP cells. (a) CCK-8 results show that HCT116/L-OHP cells were more resistant to oxaliplatin than sensitive HCT116 cells. (b) The results show that the IC10 dosage in HCT116/L-OHP cells was 20 *μ*g/mL. (c) HCT116/L-OHP cells were treated with different concentrations of oxaliplatin for 48 hours, and the inhibitory effect of ZJW combined with L-OHP on HCT116/L-OHP cells was stronger than that of L-OHP alone. (d and e) Flow cytometry results show the apoptosis rates of HCT116/L-OHP cells treated with ZJW, L-OHP, or L-OHP combined with different concentrations of ZJW. ^*∗∗*^, *P* < 0.01; ^*∗∗*^^*∗*^, *P* < 0.001 compared to HCT116/L-OHP.

**Figure 2:**
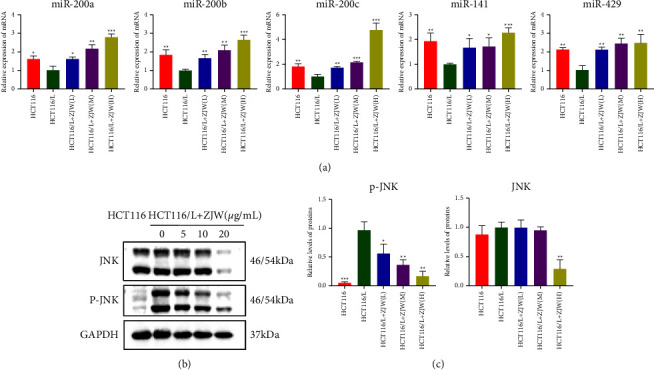
ZJW mediates the miR-200s/JNK signaling pathway to regulate drug resistance in colorectal cancer. (a) Real-time PCR assay of miR-200s levels in HCT116 and HCT116/L-OHP cells treated with L-OHP combined with different concentrations of ZJW. (b and c) Western blot assay of JNK and p-JNK protein in HCT116 cells and HCT116/L-OHP cells treated with L-OHP combined with different concentrations of ZJW. ^*∗*^, *P* < 0.05; ^*∗∗*^, *P* < 0.01; ^*∗∗∗*^, *P* < 0.001 compared to HCT116/L.

**Figure 3:**
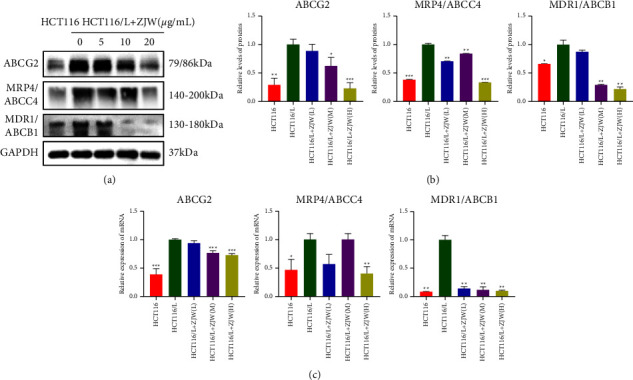
Effect of ZJW on the expression of the drug resistance-related proteins MDR1/ABCB1, MRP4/ABCC4, and ABCG2 in HCT116/L-OHP cells. (a and b) Western blot assay of ABCG2, MDR1/ABCB1, and MRP4/ABCC4 proteins in HCT116 cells and HCT116/L-OHP cells treated with L-OHP combined with different concentrations of ZJW. (c) Real-time PCR assay of ABCG2, MDR1/ABCB1, and MRP4/ABCC4 levels in HCT116 and HCT116/L-OHP cells treated with L-OHP combined with different concentrations of ZJW. ^*∗*^, *P* < 0.05; ^*∗∗*^, *P* < 0.01; ^*∗∗∗*^, *P* < 0.001 compared to HCT116/L.

**Figure 4:**
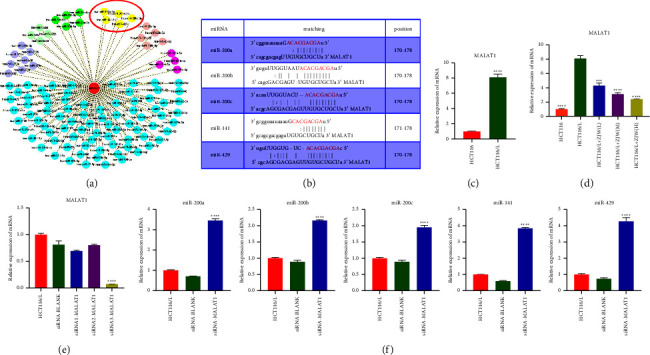
ZJW mediated the MALAT1/miR-200s/JNK signaling pathway regulating drug resistance in colorectal cancer. (a and b) Bioinformatics analysis predicted the existence of multiple complementary binding sites between MALAT1 and miR-200s. (c) Real-time PCR assay of MALAT1 levels in HCT116 and HCT116/L-OHP cells. ^*∗∗∗∗*^, *P* < 0.0001 compared to HCT116 cells. (d) Real-time PCR assay of MALAT1 levels in HCT116/L-OHP cells treated with L-OHP combined with different concentrations of ZJW. ^*∗∗∗*^, *P* < 0.001; ^*∗∗∗∗*^, *P* < 0.0001 compared to HCT116/L. (e) MALAT1 expression in control or MALAT1 siRNA-transfected HCT116/L-OHP cells. ^*∗∗∗∗*^, *P* < 0.0001 compared to HCT116/L cells. (f) miR-200s expression in control or MALAT1 siRNA-transfected HCT116/L-OHP cells. ^*∗∗∗∗*^, *P* < 0.0001 compared to HCT116/L cells.

**Figure 5:**
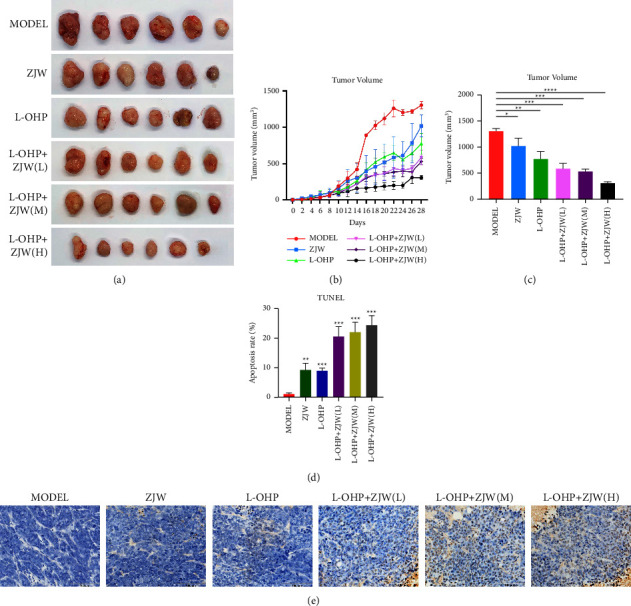
ZJW reverses MDR and affects apoptosis in a nude mouse xenograft model. (a) Photographs of nude mouse tumors in the xenograft model. (b and c) Change in tumor weight in nude mice at 28 days. (d and e) ZJW affected apoptosis in a nude mouse xenograft model. ^*∗*^, *P* < 0.05; ^*∗∗*^, *P* < 0.01; ^*∗∗∗*^, *P* < 0.001; ^*∗∗∗∗*^, *P* < 0.0001 compared to MODEL.

**Figure 6:**
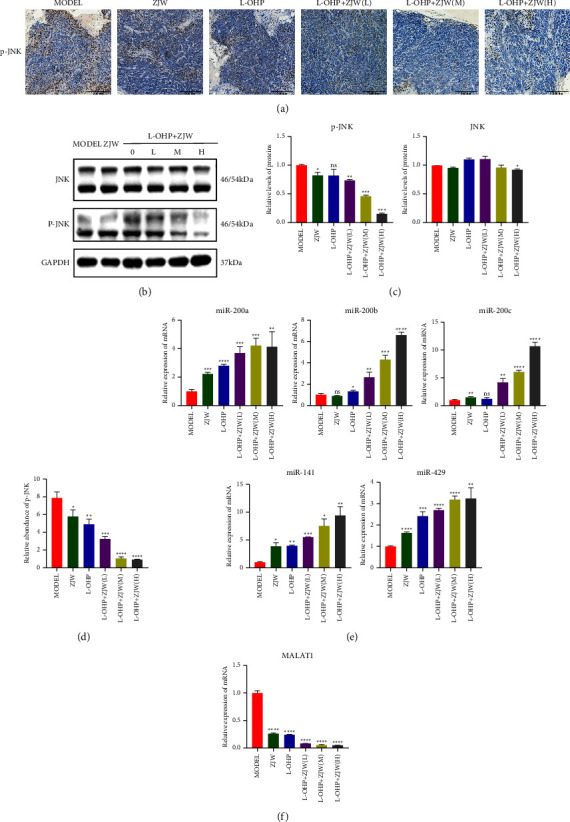
Effects of ZJW on the JNK signaling pathway, miR-200s, and MALAT1 expression in vivo. (a and d) Immunohistochemistry detection of MRP4 protein in vivo. (b and c) Western blot and real-time PCR assays of JNK and p-JNK levels in vivo. (e and f) Real-time PCR assay of miR-200s levels in vivo. (g) Real-time PCR assay of MALAT1 levels in vivo. ^*∗*^, *P* < 0.05; ^*∗∗*^, *P* < 0.01; ^*∗∗∗*^, *P* < 0.001; ^*∗∗∗∗*^, *P* < 0.0001; ns, no significance compared to MODEL.

**Figure 7:**
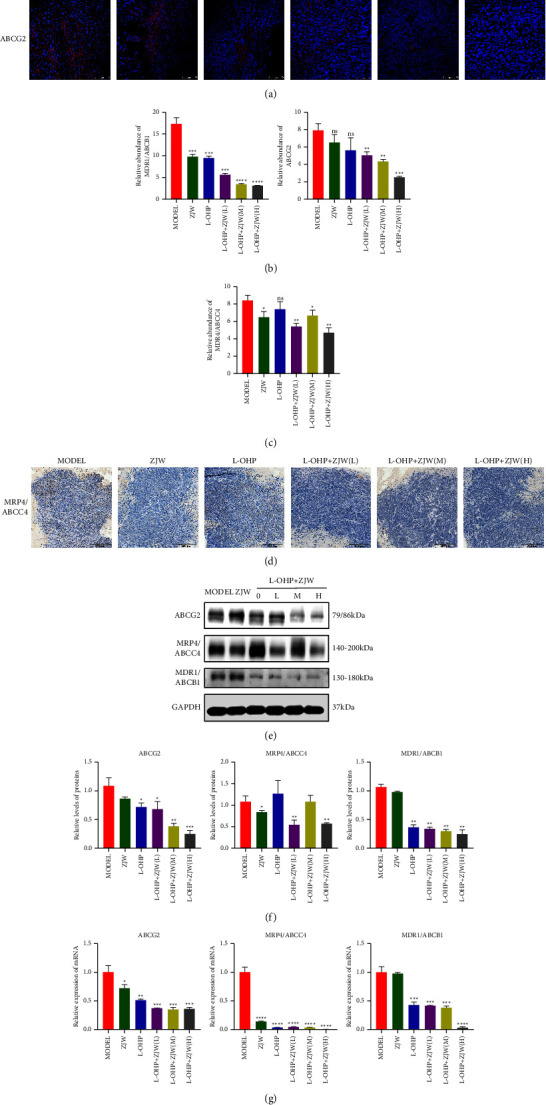
Effect of ZJW on the expression of drug resistance-related proteins in vivo. (a and b) Immunofluorescence detection of MDR1/ABCB1 and ABCG2 proteins in vivo. (c and d) Immunohistochemistry detection of MRP4/ABCC4 protein in vivo. (e and f) Western blot assay of ABCG2, MDR1/ABCB1, and MRP4/ABCC4 levels in vivo. (g) Real-time PCR assay of ABCG2, MDR1/ABCB1, and MRP4/ABCC4 levels in vivo. ^*∗*^, *P* < 0.05; ^*∗∗*^, *P* < 0.01; ^*∗∗∗*^, *P* < 0.001; ^*∗∗∗∗*^, *P* < 0.0001; ns, no significance compared to MODEL.

**Table 1:** Primer sequences.

Gene	Primer orientation	Primer sequence (5′ to 3′)
MALAT1	Forward	GCTCTGTGGTGTGGGATTGA
	Reverse	GTGGCAAAATGGCGGACTTT
ABCG2	Forward	GCAGCAGGTCAGAGTGTGGTTTC
	Reverse	ACTGAAGCCATGACAGCCAAGATG
MDR1/ABCB1	Forward	GATTGCTCACCGCCTGTCCAC
	Reverse	CGTGCCATGCTCCTTGACTCTG
MRP4/ABCC4	Forward	ATGTTCATGGGCTCCCTGTTCAAC
	Reverse	GGATGATGATGGCGGCGATGG
GAPDH	Forward	GGTGGTCTCCTCTGACTTCAACA
	Reverse	CCAAATTCGTTGTCATACCAGGAAATG
miR-200a	Forward	CCGTAACACTGTCTGGTAACGATGT
miR-200b	Forward	CGCCTAATACTGCCTGGTAATGATGA
miR-200c	Forward	CCTAATACTGCCGGGTAATGATGGA
miR-141	Forward	CGCTAACACTGTCTGGTAAAGATGG
miR-429	Forward	CCGCTAATACTGTCTGGTAAAACCGT
U6	Forward	ATGGACTATCATATGCTTACCGTA

## Data Availability

The datasets generated for this study are available on request to the corresponding author.

## Abbreviations

L-OHP: Oxaliplatin
CRC: Colorectal cancer
ZJW: Zuo Jin Wan
JNK: c-Jun N-terminal kinase
P-gp: P-glycoprotein
LncRNA: Long-stranded noncoding RNA
PTEN: Phosphatase and TENsin homolog
OSCC: Oral squamous cell carcinoma
PDCD4: Programmed cell death 4
ABCG2: ATP binding cassette subfamily G member 2
MDR1/ABCB1: ATP binding cassette subfamily B member 1
MRP4/ABCC4: ATP binding cassette subfamily C member 4.
